# Successful treatment of ivermectin refractory demodicosis with isotretinoin and permethrin cream

**DOI:** 10.1016/j.jdcr.2022.06.017

**Published:** 2022-07-01

**Authors:** Anon Paichitrojjana, Anand Paichitrojjana

**Affiliations:** a School of Anti-Aging and Regenerative Medicine, Mae Fah Luang University, Bangkok, Thailand; b Faculty of Medicine, Ramathibodi Hospital, Mahidol University, Bangkok, Thailand

**Keywords:** *Demodex* mite, demodicosis, isotretinoin, ivermectin, permethrin, SSSB, standardized skin surface biopsy

## Introduction

Demodicosis is a chronic inflammatory skin disease caused by *Demodex* mites. Abnormal proliferation and density of *Demodex* mites are essential factors for the occurrence of skin disorders.^1^ There are many clinical manifestations of demodicosis, such as pityriasis folliculorum type, rosacea-like type, perioral dermatitis–like type, and folliculitis or acne-like type.^2^ Diagnostic criteria for demodicosis should be based on a suspected skin lesion, confirmed by the abnormal density of *Demodex* mites and clinical improvement after acaricidal treatment. Several reports have documented the successful treatment of demodicosis with ivermectin and acaricidal agents, but there has never been a report of *Demodex* mite’s refractory to ivermectin.^3^ We present a patient with ivermectin refractory demodicosis, successfully treated with oral isotretinoin combined with permethrin.

## Case report

**Fig 1:**
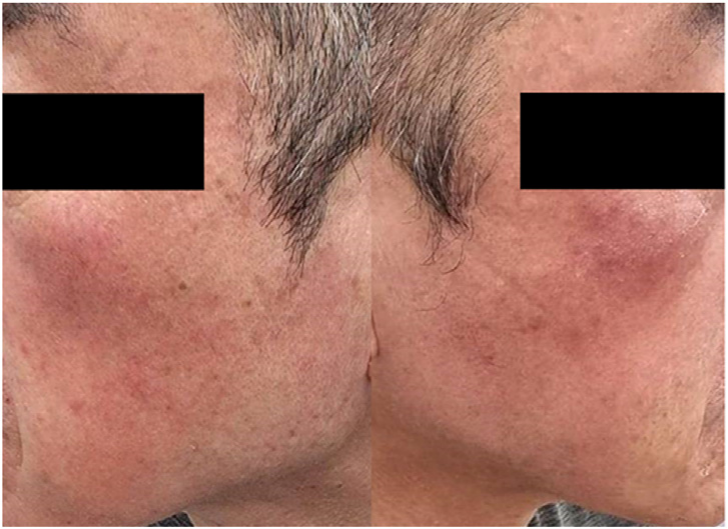
Dry, scaly, erythematous patches and telangiectasia with a stinging and burning sensation on both cheeks.

**Fig 2:**
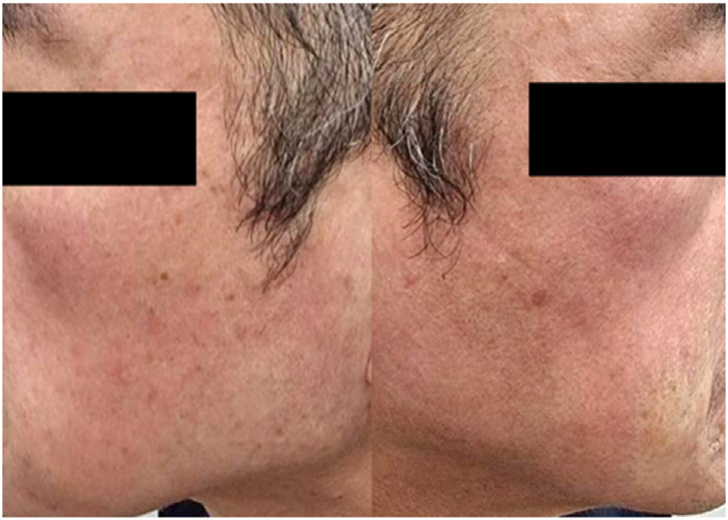
Complete resolution of all clinical symptoms after 8 weeks of treatment with isotretinoin and permethrin cream.

A healthy 63-year-old man presented with dry, scaly, erythematous patches and telangiectasia with a stinging and burning sensation on both cheeks (Fig 1). He also had a history of itchy bumps resembling insect bites on his face, which spontaneously disappeared in a few days. Two years ago, he was diagnosed with demodicosis and treated successfully with 2 doses of oral ivermectin (200 μg/kg) in combination with metronidazole gel 0.75%. Approximately 10 months ago, he developed a scaly, eczematous rash with irritation on his face and was treated with triamcinolone cream 0.1%. The rash improved, but after stopping topical medication for a few weeks, the rash reappeared at the same location. Despite continued treatment, his clinical course slowly deteriorated with the development of more stinging, burning sensation and telangiectasia on both cheeks that no longer responded to topical steroids. At this time, the standardized skin surface biopsy (SSSB) was performed on the rash, revealing numerous *Demodex* mites (68 mites/cm^2^). He was diagnosed with demodicosis and treated with 2 doses of oral ivermectin (200 μg/kg, 1 week apart) in combination with metronidazole gel 0.75%. This therapy resulted in a slight improvement of the clinical lesions at the 2-week follow-up, so ivermectin treatment was continued for another 4 doses. At the 1-month follow-up, there was some improvement with reductions in skin hypersensitivity and telangiectasia. However, the dry, scaly erythematous patches and some papules persisted. SSSB revealed 61 *Demodex* mites/cm^2^. Ivermectin and metronidazole gel were discontinued. A trial of low-dose oral isotretinoin 10 mg, 4 times a week (0.1 mg/kg/day) combined with permethrin cream 1% was administered for another 8 weeks. This therapy resulted in marked improvement of the lesions (Fig 2). There was a complete resolution of all clinical symptoms, and repeat SSSB confirmed the eradication of *Demodex* mites, defined by the consecutive negative results with less than 5 *Demodex* mites/cm^2^ spanning beyond 2 mite-life cycles. The isotretinoin treatment and permethrin cream were continued for 6 months. There was no recurrence at 9 months after isotretinoin administration.

## Discussion

Rosacea and rosacea-like demodicosis have many similarities. It is still controversial in the literature that erythematotelangiectatic rosacea possibly corresponds to a subclinical stage of demodicosis.^4^ From our point of view, although this patient had an erythematous patch with telangiectasia like those seen in rosacea, the dry, scaly erythematous patch with a stinging and burning sensation is more compatible with demodicosis. In addition, the severity of the patient's symptoms was related to the number of *Demodex* mites, and all clinical symptoms disappeared after the eradication of *Demodex* mites.

Ivermectin is a broad-spectrum antiparasitic drug that reaches peak plasma levels 5 hours after oral administration. It has a half-life of 36 hours, while the peak concentration in the squames, sebum, and sweat was 8 hours.^5^^,^^6^ Ivermectin’s mechanism of action is to block chemical transmission across the nerve synapses that use glutamate-gated anion channels or γ-aminobutyric acid–gated chloride channels causing paralysis of nematodes and arthropods.^7^ Ivermectin is harmless in humans because these specific targets are found only in the central nervous system, while ivermectin cannot penetrate through the blood-brain barrier.

There are no standardized therapeutic regimens of ivermectin for demodicosis; the effective dosage reported in the literature is a single oral dose of 200 μg/kg, but sometimes the regimen has been reported to be repeated doses every 1 or 2 weeks for 2-3 times.^8^ A second oral dose of ivermectin 1 week after the initial dosage is a more reasonable treatment regimen because the larvae of *Demodex* mite hatch from the egg in 3-4 days and develop into adult form in about 7 days, while ivermectin does not have an ovicidal effect and the plasma half-time is only 36 hours.^5^ Therefore, it is difficult to give an appropriate definition of ivermectin resistance. This patient was treated with ivermectin once a week for 6 weeks without significant clinical improvement. Moreover, an abnormally high number of *Demodex* mites were still detected in the skin lesions. Oral ivermectin and metronidazole gel failed to reduce the number of *Demodex* mites and relieve *Demodex*-related symptoms. Thus, *Demodex* mites from this patient are refractory to the treatment with ivermectin and metronidazole gel. The patient is healthy and does not take any immunosuppressive drugs. The patient's spouse had no clinical symptoms of demodicosis, and the *Demodex* mites could not be detected from her face by SSSB, so a reinfection from the spouse is unlikely. There are 2 possible mechanisms of ivermectin refractory or resistance. The first is an alteration of P-glycoprotein, a membrane protein that transports ivermectin across cell membranes, and the second is the modification of the chloride channel receptor.^9^

The author used isotretinoin in this patient because it reduces sebaceous gland size and suppresses sebum production, which are the habitat and food sources of *Demodex* mites.^10^ Furthermore, isotretinoin also shows anti-inflammatory effects by inhibiting the migration of polymorphonuclear leukocytes into the skin and downregulating Toll-like receptor 2 expressions. Low-dose isotretinoin avoids side effects, especially drying effects on the skin, since patients with demodicosis are more likely to experience stinging, burning, and irritation. Combination therapy with permethrin cream 1% as it had an acaricidal effect and minor skin irritation than permethrin cream 5%. Clinical improvement and negative results of SSSB in our patient started 8 weeks after the combination treatment. The successful treatment of this patient with isotretinoin and permethrin cream was likely due to isotretinoin supporting the acaricidal effect of permethrin cream, resulting in the eradication of *Demodex* mite and a complete resolution of all clinical symptoms.

This is a case report of ivermectin refractory demodicosis successfully treated with isotretinoin and permethrin cream. Ivermectin refractory of *Demodex* mites should be considered in recurrence or recalcitrant demodicosis. The effectiveness of demodicosis treatment with isotretinoin and topical acaricidal agents needs to be assessed in prospective controlled clinical trials with long-term follow-up.

## Statement of ethics

The author states that the patient gave written informed consent for the case to be published (including publication of images). This research complies with all Ethical Guidelines for human studies in accordance with the World Medical Association Declaration of Helsinki. This paper is exempt from The Mae Fah Luang Ethics Committee on Human Research approval, with reference number COE 072/2022. Since it is a case report with no more than 3 cases, the information is derived from a review of medical records and cannot be linked to an individual unless the written consent of the patient is obtained.

## Conflict of interest

None disclosed.
